# Retention of children under 18 months testing HIV positive in care in Swaziland: a retrospective study

**DOI:** 10.11604/pamj.2017.28.316.13857

**Published:** 2017-12-26

**Authors:** Nomvuselelo Sikhondze, Ozayr Haroon Mahomed

**Affiliations:** 1 Discipline of Public Health Medicine, University of KwaZulu Natal, Durban, South Africa

**Keywords:** Retention into care, antiretroviral treatment, Swaziland

## Abstract

**Introduction:**

Significant progress has been made with respect to the initiation of children on antiretroviral therapy (ART) in Southern Africa including Swaziland, however retention of these children in care poses a major challenge. The aim of the study was to assess retention to care in children testing HIV positive taking into account the number of return child welfare care (CWC) visits the child made.

**Methods:**

A retrospective cross sectional study and was conducted at 4 facilities in Swaziland. All children who were HIV infected from 0 to 18 months were identified using the child welfare register (CWC). Infant characteristics were obtained from the child welfare register and early infant diagnosis logbooks. Proportion of patients retained in care were calculated at three, six, nine and twelve months.

**Results:**

Of the 32 HIV positive children identified tested between December 2014 up to July 2016, sixty eight percent (n = 22) of the children that tested HIV positive were retained at three months, 40.6% at six months, 18.8% at nine months and 12.5% at twelve months. Children that resided in urban areas, more male than female children, children from mothers who were on antiretroviral treatment, children initiated on antiretroviral treatment, mothers on antiretroviral treatment for more than one year and children who received Infant Nevirapine were more likely to be retained.

**Conclusion:**

Facilities are performing well in terms of identifying HIV positive children within the first two months of life and linking them into care. However, as time progresses the retention of children in care declines. Innovative strategies need to be developed to enhance patient retention.

## Introduction

Human Immune Deficiency Virus (HIV) continues to be a major global public health issue. In 2015, there were 36.7 million (30.8 million-42.9 million) people living with HIV globally, including 2.1 million (1.7 million-2.6 million) children (< 15 years) [1]. East and Southern Africa were the most affected region with 960,000 new HIV infections, representing 46% of the global burden [2]. About 150 000 (110 000-190 000) children became infected with HIV in 2015 [3]. Swaziland, a small landlocked country in southern Africa, has the highest HIV prevalence in the world, with 28.8% of their adult population living with HIV. The epidemic is generalised affecting all regions. The HIV prevalence in 2016 was 27% in Hhohho, 30% in Manzini, 28% in Shiselweni and 30% in Lubombo [4]. In 2015, 11,000 people were newly infected with HIV and 3,800 people died of an AIDS-related illness [5]. The HIV incidence trends amongst adults between the age of 15 and 49 years has declined steadily from 2, 23 per 100 person years in 2011 to 1, 85 per 100 patient years in 2016 [6] as a result of the concerted effort of the Swaziland Government to curb the HIV epidemic. Approximately 144,412 (64%) of people living with HIV were on anti-retroviral treatment (ART) in 2016 [7]. Pregnant women are a high risk group for the acquisition and transmission of HIV. More than 97% of antenatal attendees in Swaziland were aware of their HIV status, with approximately 35% of all pregnant women (10560) testing HIV positive in 2014. Ninety percent (9,491) of eligible pregnant women received ART in 2014 [8], with 99% of the exposed infants receiving Cotrimoxzole prophylaxix. In terms of the Swaziland integrated HIV management guidelines (2015) [1], all HIV exposed infants should be provided with Nevirapine and Cotrimoxazole prophylaxis for 6 weeks and the first HIV DNA PCR test should be conducted. If the infant is HIV positive then linkages for ART should be established. However, if the infant is negative, the infant should be continued with Cotrimoxazole until breastfeeding has been stopped and HIV has been excluded. The purpose of this study was to determine the proportion of children less than 18 months that tested HIV positive who were retained in care. Retention in care was defined as the patient attending the prescribed follow up child welfare visit (3 months (14 weeks), 6 months, 9 months, 12 months, 15 months and 18 months) as defined by the Swaziland integrated HIV management guidelines (2015) [1] post-natal care schedule.

## Methods

**Study design and setting**: A retrospective study and was conducted in 4 facilities in the country namely Mbabane Public Health Unit, Lobamba clinic, Ezulwini Satellite clinic and Hhukwini clinic. Mbabane PHU is a Primary health care facility situated in Mbabane City next to Mbabane Hospital in the Hhohho region. The facility serves a catchment population of an average of 4,700 per year and an average of 45 exposed children at first child welfare visit i.e. at 6 to 8 weeks visit, per month. Lobamba and Ezulwini satellite clinics are situated in the semi urban community and serves a catchment population of 12,000. On average these facilities see 25 exposed children per month at first child welfare care visit. Hhukwini is situated in the rural area and serves a catchment population of 5,718 and sees and average of 4 exposed children per month at first child welfare care visit.

**Study population**: The study population included all HIV positive children testing positive who were below 18 months during the course of the data collection.

**Data collection**: All children who were HIV infected from 0 to 18 months were identified using the child welfare register (CWC). Infant and maternal characteristics were obtained from the CWC Register and early infant diagnosis logbooks. Data on the number of child welfare visits and the duration between testing and initiation of ART were obtained from the patients clinical records. Reasons for not initiating ART were obtained from the CWC registers. Proportion of patients that were retained in care at three months, six months, nine months and twelve months that were HIV exposed were calculated.

**Ethics**: The study was approved by the Bio Medical Research Ethics Committee of the University of KwaZulu Natal (BE 385/16). Permission to conduct the study was obtained from the Swaziland Ministry of Health. Patients name and addresses were omitted from the data extraction tool to maintain patient confidentiality and privacy.

## Results

**Demographic characteristics of respondents**: Thirty two HIV positive children identified from four health facilities in Swaziland, with 75% from two facilities (Mbabane Public Health Unit (n = 12) and Lobamba Clinic (n = 12)). Seventy eight percent (n = 25) of the children resided in rural areas with most of the HIV positive children were male (56.3%; n = 18) (Table 1).

**Table 1:** Frequency table of the demographic characteristics of the study population (2015)

		**n**	**%**
Health Facility Name	Ezulwini Satellite Clinic	6	18.8
	Hhukwini Clinic	2	6.3
	Lobamba Clinic	12	37.5
	Mbabane PHU	12	37.5
Residence	Rural	25	78.1
	Urban	7	21.9
Gender	Female	14	43.8
	Male	18	56.3

**Characteristics of mothers**: Seventy eight percent (n = 25) of the children who tested positive were born from mothers already on antiretroviral treatment with (92%, n = 23) of the women initiating antiretroviral treatment during pregnancy at ANC and 8% (n = 2) initiated after pregnancy at post-natal care. More than half (52%) of the women initiated antiretroviral treatment in 2015 (52%, n = 13) whilst about 40% (n = 10) initiated before 2015 (Table 2).

**Table 2:** Frequency table of the characteristics of mothers of infants

		**n**	**%**
Mother initiated on ART	No	7	21.9
	Yes	25	78.1
Place initiated	ANC	23	92.0
	Post-natal	2	8.0
Year initiated	Before 2015	10	40.0
	2015	13	52.0
	2016	2	8.0

**Early infant diagnosis for HIV**: All 32 children received a confirmatory HIV test, with 94% (n = 30) receiving their HIV test results. Seventy two percent (n = 23) were diagnosed within the recommended 6 to 8 weeks and were referred for ART. Of all the children, more than half of the children (62%, n = 20) were initiated on ART whilst 38% (n = 12) were not initiated. Of the 32 children identified to be HIV positive 81% (n = 26) were on Infant Nevaripine prophylaxis (I-NVP) for the first 6 weeks of life and 19% (n = 6) were not initiated on INVP (Table 3). The main reason for the children not been initiated on ART was that the patients did not return for child welfare visits (83%, n = 10) and 17% (n = 2) had died before initiation 8.3% (n = 1) (Table 3).

**Table 3:** HIV testing and ART Initiation

		**n**	**%**
Received a confirmatory Test DNA or PCR	Yes	32	100
Received HIV test result	No	2	6.3
	Yes	30	93.8
Referred for ART	No	8	25
	Yes	23	71.9
	Missing	1	3.1
Initiated on ART	No	12	37.5
	Yes	20	62.5
Reason for not initiating on ART	Loss to follow up	10	83%
	Died before initiation	2	17%

**Figure 1:**
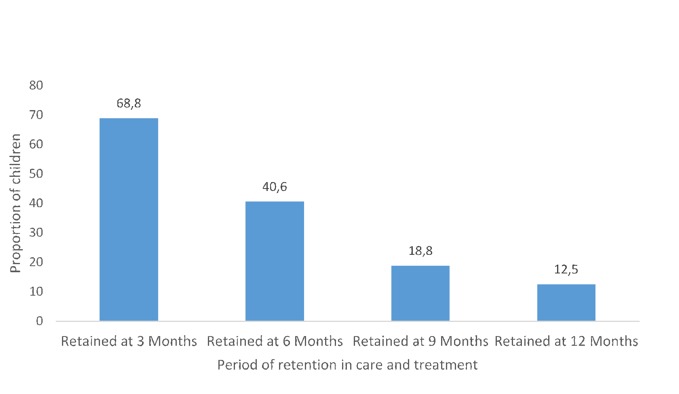
Proportion of HIV positive children retained in HIV care and treatment between three and eight months

**Retention in HIV care and treatment**: Sixty eight percent (n = 22) of the children that tested HIV positive were retained at 3 months, 40.6% (n = 13) at 6 months, 18.8% (n = 6) at 9 months and 12.5% (n = 4) at 12 months (Figure 1).

**Retention in HIV Care and treatment at 3 months**:Of the 22 children 100% of urban children and 60% (n = 15; N = 25) of the children that came from rural areas were retained at 3 months. Seventy one percent (n = 10; N = 14) of male children compared to 66.7 % (n = 12; N = 18) of female children, 80% (n = 20; N = 25) of children from mothers who were on ART, 80% (16) of children who were initiated on ART and 73% (19) of the children that received Nevirapine prophylaxis were retained at 3 months (Table 4)

**Table 4:** Frequency table of profile of children retained at three months

	**n**	**%**
Residence- Rural	15 (25)	60%
Residence - Urban	7 (7)	100%
Male	10 (14)	71%
Female	12 (18)	67%
Mother initiated ART	20 (25)	80%
Year mother initiated ART		
Before 2015	10	100%
2015	10	77%
2016	0	0%
Child referred for ART	16 (20)	80%
Child Initiated on ART	16 (20)	80%
Received INVP Prophylaxis	19 (25)	73%

## Discussion

The findings from this study, despite the small number of patient's highlights strengths and programmatic weaknesses within the PMTCT programme in Swaziland. The strengths of the PMTCT programme demonstrated through the results of the study include amongst others is that there was adherence to the World Health Organisation recommendations that infants exposed to HIV should be tested at 4-6 weeks of age, using a virological test Dried blood spot to polymerase chain reaction testing and ART should be started as soon as the infants are found to be HIV-positive, regardless of clinical and immune status [9]. In this study all children received a confirmatory HIV test within 6 weeks, 72% (n = 22) were diagnosed within the recommended 1.5 to 2 months and (62%, n = 20) being initiated on ART. This represents an improvement in 2010 only 47 per cent of HIV-exposed infants received an HIV test at six to eight weeks of age; 78 per cent of exposed infants received co-trimoxazole; only 28 per cent of HIV-infected infants received ART; and exclusive breastfeeding rates are at 44 per cent for infants less than six months old [10]. Loss to follow up (83%) and infant death (17%) were the main reason for non-initiation of infants on ART. Amongst the contributing factors to the loss to follow up was the lag in time between HIV testing and the parents/caregivers receiving results (1.2 months) and the time from receiving results to initiating the children on ART was 2 months. The quality of care provided to HIV exposed infants was sub-optimal as 19% (6) of the infants were not initiated on Infant Nevirapine, which was inconsistent with the WHO HIV management guidelines that requires all children born to HIV positive mothers should be prescribed Infant Nevirapine (INVP) for the first six weeks of life as prophylaxis to strengthen prevention of mother to child transmission of HIV (MTCT) [11]. The proportion of patients retained in care declined from 68.8% at three months to 12.5% after 12 months of care. These findings are much lower than the findings a study conducted in Fort Portal, Western Uganda, to assess adherence to Option B+ until 18 months postpartum that showed 82.1% retention at six months and 71.6% retention at 12 months [12]. Furthermore, the findings of the current study are contrary to the findings from a systematic review that included 31877 children, all from African countries which showed a total of 5,558 (19%) children were not retained: 4082 (73%) were reported loss to follow up and 1476 (27%) died [13]. Amongst the African countries that have reported on paediatric patient retention, Rwanda has the highest retention (95%) [14] and the lowest retention is in a West African cohort (71%) [15].

In Swaziland the drop in patient retention on the programme could be attributed to stigma and discrimination driven by community norms, myths and misconceptions that do not support HIV prevention efforts. Furthermore, health worker attitudes towards HIV-positive mothers, especially at labour and delivery and early post-natal care serve to enhance the myths resulting in patients withdrawing from care [10]. Patients that withdraw from care or are not adherent to medication are not followed up due to the weak linkages and referral mechanisms between community and facility based services [10]. The characteristics of the patients retained at three months suggest that children that that resided in urban areas, more male than female children, children from mothers who were on ART, children initiated on ART, mothers on ART treatment for more than one year and children who received Infant Nevirapine were more likely to be retained. These findings suggest that loss to follow up depend on the child's caregivers and are impacted by social determinants of health such as level of education, income level, availability of transportation, access to health services and living conditions [13]. Of concern, in this study seventy eight percent (n = 25) of the children who tested positive were born from mothers already on antiretroviral treatment with (92%, n = 23) of the women initiating antiretroviral treatment during pregnancy at antenatal care. This is an indication of probably the late initiation of antenatal care and ART uptake. According to the PMTCT guidelines, the ARV regimen should be initiated as early as possible after 14 weeks, which is only possible if the pregnant woman visits antenatal care within the first trimester [1]. Secondly, this could be due to the poor adherence to treatment of patients on ART- as the maternal, child health programme have poor capability for tracking clients and ensuring that all services are received and women adhere to their drug regimens throughout their pregnancy [10].

**Study Limitations**: The study was affected by the limited number of patients enrolled within the study over the period of the study. Data for the children beyond twelve months was not available.

## Conclusion

Attrition from paediatric care and treatment programs has severe consequences on morbidity and mortality. The findings of this study indicate that children who are identified to be HIV positive are linked to care, however retention in care is a major challenge. There is a need to harmonize the information system in terms of improving the documentation in the child welfare registers and also improving the referral and linkages between the facility and outreach teams. Innovative mobile technology should be utilised for health promotion and reminder messages.

### What is known about this topic

- Pregnant women are a high risk group for the acquisition and transmission of HIV;
- More than 97% of antenatal attendees in Swaziland were aware of their HIV status, with approximately 35% of all pregnant women testing HIV positive in 2014;
- Ninety percent of eligible pregnant women received ART in 2014, with 99% of the exposed infants receiving Cotrimoxazole prophylaxis.

### What this study adds

- Retention in care in Swaziland has declined over the observation periods;
- Provides a baseline for further studies to investigate factors associated with poor retention in care.

## Competing interests

The authors declare no competing interests.
